# Missouri Veterinary Medical Association

**Published:** 1900-11

**Authors:** B. F. Kaupp

**Affiliations:** Secretary


Secretary.

### MISSOURI VETERINARY MEDICAL ASSOCIATION.

THE ninth annual meeting of this State Association opened at 9 A.M., October 3, 1900, in the hall of the Board of Education Building, St. Louis, Mo. The following veterinarians were present: Drs. E. J. Netherton, W. B. Welch, R. H. Carswell, T. V. Farmer, S. L. Shaw, E. Lee, S. Stewart, L. D. Brown, Charles Ellis, Charles Doerrie, T. E. White, V. J. Andre, H. Hempleman, R. A. Kammerer, W. S. Cass, F. W. O'Brien, W. E. Martin, T. F. Arnold, T. J. Menastrina, L. M. Klutts, D. W. Elliott, S. E. Phillips, R. C. Moore, H. Bradley, J. M. Phillips, B. F. Kaupp, G. A. Roberts, H. B. Piatt, M. McNally, E. J. Forman, D. C. Barnett, J. O. F. Price, W. H. Meadors, T. B. Pote, W. F. Heyde, C. Miller, J. W. Connaway, J. G. Slee. Among the guests of the Association were H. J. Waters, Dean and Director of the Agricultural College and Experiment Station of Missouri; Alex. Maitland, member of the State Board of Agriculture, and others.

The following veterinarians made application and were elected to membership: Drs. V. J. Andre, R. H. Carswell, T. J. Menestrina, E. J. Netherton, L. D. Brown, S. L. Shaw, E. B. Shaw, and D. F. Luckey.

The morning session was devoted to the discussion of State legislation. A bill was drafted and a committee appointed to push it in the next session of the State Legislature. A committee from the Missouri Valley Veterinary Association was accepted to assist this committee. At 12 o'clock it was moved, seconded, and carried to adjourn to visit the St. Louis Dressed Beef and Provision Co.'s plant under the direction of Dr. W. S. Cass, where the guests were afforded the opportunity to observe the methods of government inspection of cattle, hogs, sheep, and calves and their methods of stamping and tagging such meat products. A few interesting cases which had been condemned were examined, and many other things of interest were seen. At 1.30 P.M. the party was conducted to the Delmonico, at Manchester Road and King's Highway, where they were banqueted in royal style by the veterinarians of St. Louis, Dr. Charles Ellis acting as toastmaster.

At 3 P.M. a surgical clinic was held at Dr. R. A. Kammerer's hospital. Dr. R. C. Moore, of Kansas City, demonstrated a new method of administering chloroform by a specially arranged nose-bag, and performed the operation of median neurectomy. An operation by Dr. E. J. Netherton upon a carcinomatous growth affecting the eye of a horse. Dr. Moore, assisted by Drs. Ellis and Andre, performed the operation of arytenoiderrhaphy upon a "roarer." The operation of tenotomy for "stringhalt" and several other operations and clinical cases to be diagnosed were presented, which lasted until 6 P.M.

At 7.30 P.M. the meeting was again called to order, when the following papers were presented: Dr. H. Bradley, "Acute Bowel Trouble;" Dr.

Charles Ellis, "Needed Veterinary Legislation in Missouri;" Dr. E. Brainard, "Influenza;" Dr. T. J. Menestrina, "Canine Distemper;" Dr. J. W. Connaway, "Parturient Paresis in the Cow." Under reports of cases Dr. J. M. Phillips reported an interesting and unusual case in which the teeth in a thirteen-year-old dog became very loose, making extraction necessary. Some months later three new teeth developed. At 11.30 P.M. the meeting adjourned.

At 9 A.M., October 4th, the meeting was again called to order by the President, Dr. Bradley, when the following papers were offered: Dr. C. Miller, "Infectious Ulcer of the Vulva of Cattle;" Dr. J. W. Parker, "A Bacillus from an Infectious Vulvar Disease of Cattle;" Dr. S. Stewart gave a good and interesting account of the last meeting of the American Veterinary Medical Association; Dr. L. M. Klutts, "Laminitis;" Dr. F. W. O'Brien, "My Experience with Schmidt's Treatment of Parturient Apoplexy."

The following officers were elected for the coming year: President, Dr. F.W. O'Brien; Vice-President, Dr. J. W. Connaway; Secretary-Treasurer, Dr. B. F. Kaupp. A motion was made, seconded, and carried to hold the next meeting in Kansas City in October, 1901.

At 12 o'clock the meeting adjourned, and after luncheon the party proceeded to the St. Louis Fair, where an enjoyable afternoon was spent. About dark the party disbanded, ending the most successful meeting in the history of the Association.

B. F. KAUPP, D.V.S.,
Secretary.

---

THE annual meeting of the Illinois State Veterinary Medical Association will be held at the Sherman House, Chicago, November 14 and 15, 1900. Every veterinary surgeon in the State is invited to attend this meeting. A series of surgical clinics will be held each day. Practitioners having papers to read at this meeting will please notify either of the undersigned.

W. J. MARTIN, President,

Kankakee, Ill.

A. C. WORMS, Secretary,

354 Racine Ave., Chicago, Ill.

THE next meeting of the Veterinary Medical Association of New Jersey will be held in Trenton, January 10, 1901.

